# Preoperative Prediction of Microvascular Invasion of Hepatocellular Carcinoma: Radiomics Algorithm Based on Ultrasound Original Radio Frequency Signals

**DOI:** 10.3389/fonc.2019.01203

**Published:** 2019-11-14

**Authors:** Yi Dong, Qing-Min Wang, Qian Li, Le-Yin Li, Qi Zhang, Zhao Yao, Meng Dai, Jinhua Yu, Wen-Ping Wang

**Affiliations:** ^1^Zhongshan Hospital, Fudan University, Shanghai, China; ^2^Department of Electronic Engineering, Fudan University, Shanghai, China; ^3^Harvard Medical School, Massachusetts General Hospital, Boston, MA, United States

**Keywords:** hepatocellular carcinoma, microvascular invasion, prediction, radiomics analysis, original radio frequency signals

## Abstract

**Background:** To evaluate the accuracy of radiomics algorithm based on original radio frequency (ORF) signals for prospective prediction of microvascular invasion (MVI) in hepatocellular carcinoma (HCC) lesions.

**Methods:** In this prospective study, we enrolled 42 inpatients diagnosed with HCC from January 2018 to December 2018. All HCC lesions were proved by surgical resection and histopathology results, including 21 lesions with MVI. Ultrasound ORF data and grayscale ultrasound images of HCC lesions were collected before operation for further radiomics analysis. Three ultrasound feature maps were calculated using signal analysis and processing (SAP) technology in first feature extraction. The diagnostic accuracy of model based on ORF signals was compared with the model based on grayscale ultrasound images.

**Results:** A total of 1,050 radiomics features were extracted from ORF signals of each HCC lesion. The performance of MVI prediction model based on ORF was better than those based on grayscale ultrasound images. The best area under curve, accuracy, sensitivity, and specificity of ultrasound radiomics in prediction of MVI were 95.01, 92.86, 85.71, and 100%, respectively.

**Conclusions:** Radiomics algorithm based on ultrasound ORF data combined with SAP technology can effectively predict MVI, which has potential clinical application value for non-invasively preoperative prediction of MVI in HCC patients.

## Introduction

Hepatocellular carcinoma (HCC) is the third leading cause of cancer-related death worldwide and the first leading cancer in East Asia (1). Resection is the most commonly used treatment for patients with early stage HCC. However, recurrence within 2 years after surgery still occurs in 30–50% of patients, which becomes the major cause of mortality (2). The early recurrence of HCC has been found to be associated with the microvascular invasion (MVI) of tumor emboli in close proximity to the primary HCC (3). MVI was proved to be an important factor not only for predicting early recurrence but also for assessing long-term patient survival (4). The presence of MVI is a histopathological indication of aggressive behavior of HCC (5), especially in the first 2 years after liver resection and transplantation (3). Both univariable and multivariable analyses revealed that MVI was independently associated with poorer overall survival rate and recurrence-free survival rate after partial hepatectomy for HCC (6). Accurate and successful preoperative assessment of MVI in patients with HCC may be helpful to make appropriate clinical management strategy, and finally, to improve survival rate of HCC patients.

At present, MVI could only be diagnosed by surgical pathology after operations and was reportedly presented in 15.0–57.1% HCC surgical specimens (5, 7). Some studies have made persistent endeavors toward the preoperative prediction of MVI (8–10). Several radiological features on contrast-enhanced magnetic resonance imaging (MRI) and computed tomography (CT) images, such as tumor margin, internal arteries, and hypodense halos, were found to be associated with MVI (11–13). However, MR or CT imaging has limitations for predicting the tumor MVI in HCC (14, 15). The reported sensitivity and specificity of preoperative prediction of MVI in HCC lesions based on contrast-enhanced CT were only 81.7 and 88.1%, respectively (16). The results of MRI showed that the mismatch between diffusion-weighted imaging (DWI) and T2-weighted imaging of regions was an independent predictor of MVI, with higher specificity (95.65%) but less sensitivity (18.18%) (14, 15). In addition, it is difficult to predict MVI in small tumors; the imaging predictors such as internal arteries and hypodense halos were not frequently observed in small tumors (8). Up till now, there is still debate about the best imaging predictive feature of MVI in HCC (11–13).

Recently, radiomics analysis based on ultrasound imaging (RA-USI) technology has achieved some good results in the early diagnosis, prognosis, and prediction of diseases (17–19). The accuracy of grading diagnosis of liver cirrhosis using RA-USI was proved to be more accurate than that of traditional ultrasound elastography technology (20). In our previous study, we also confirmed that the multiparametric ultrasound model based RA-USI achieved a good performance with mean AUC values of 0.78–0.85 (20). However, current radiomics analysis was based on conventional ultrasound images; it faced some limitations, such as influence of standardization of ultrasound images, diversity of electronic characteristics caused by different ultrasound equipment, and speckle noise of different ultrasound equipment (19).

To improve the diagnosis and treatment efficiency, original image with abundant signal information might be necessary. Comparing with conventional ultrasound images, ultrasound original radio frequency (ORF) signal is not affected by postprocess such as brightness compensation, envelope detection, depth compensation, or dynamic range adjustment (21). ORF contains all the acoustic information, including attenuation, scattering, sound speed, phase, and so on, which might provide more abundant tissue information than conventional ultrasound images (22). ORF signal would only be related to the physical transmitting and receiving mechanism of imaging equipment (23). Therefore, ORF signal contains more abundant macro- and microtissue information than conventional ultrasound images (24). It is expected to obtain higher stability and consistency in further radiomics analysis process.

In this study, we aimed to investigate the value of radiomics algorithm based on ultrasound ORF data (RA-ORF) in preoperative detection of MVI in HCC patients.

## Materials and Methods

### Patients

From January 2018 to December 2018, patients preoperatively diagnosed with HCC in a single institution were enrolled. The inclusion criteria were (1) adult patients suspected to be primary HCC by imaging methods and planned to accept surgery in our hospital; (2) solitary tumor; (3) all patients accepted preoperative grayscale ultrasound examinations within 1 week before surgery; (4) HCC lesions located in the right lobe of liver; and (5) all cases were confirmed by histopathological examination and MVI evaluation.

Exclusion criteria included the following: (1) target HCC lesion not clearly visible on the grayscale ultrasound scan; (2) patients with preoperative biopsy or adjuvant therapy (radio frequency therapy, chemotherapy, targeted therapy, etc.); (3) incomplete clinical or histopathological data; and (4) patients with HCC larger than 5 cm in maximum diameter, since such tumors are known to have a greater risk of MVI.

### Final Diagnosis

The final histopathological results including MVI grade were the gold standard for our current study. According to the practice guidelines of Chinese Society of Pathology, MVI was defined based on the number of cells that can be found in the endothelial vascular lumen under microscopy. MVI were divided into three additional subgrades, including M0, no MVI; M1 (the low-risk group), ≤5 MVI in adjacent liver tissue ≤1 cm away from the tumor; and M2 (the high-risk group), >5 MVI or MVI in liver tissue >1 cm away from the tumor (25).

Two pathologists with at least 10 years of experience in hepatic pathology reviewed all the specimen slices. Both investigators were blinded to the clinical and imaging information of the patients. In cases of discordance, a consensus reading was performed.

### Ultrasound Imaging Procedure and ORF Data Acquisition

All patients fasted for at least 8 h before ultrasound examinations. The grayscale ultrasound examinations of the hepatic lesions were performed according to the standardized protocol. Ultrasound examinations were performed by a single experienced radiologist (more than 18 years' experience of liver ultrasound scan), who was aware of the patients' clinical history. All ultrasound examinations were performed with an EPIQ-7 ultrasound system certificated with ORF data (Philips Medical Company). A C5-1 curved transducer (1–5 MHz) was used for data acquisition.

First, conventional grayscale ultrasound scan was performed. After a clear ultrasound image of tumor was obtained, the process of ORF data acquisition was started. We clicked the “freeze” button to freeze the grayscale ultrasound images and to save the current ORF data retrospectively. The corresponding conventional grayscale ultrasound images were also captured to build a comparison test for ultrasound ORF signals. Both of them would be further used to establish MVI preoperative prediction radiomics models.

### ORF Data Processing and Radiomics Analysis Procedure

#### Overall Design

**Figure 1:**
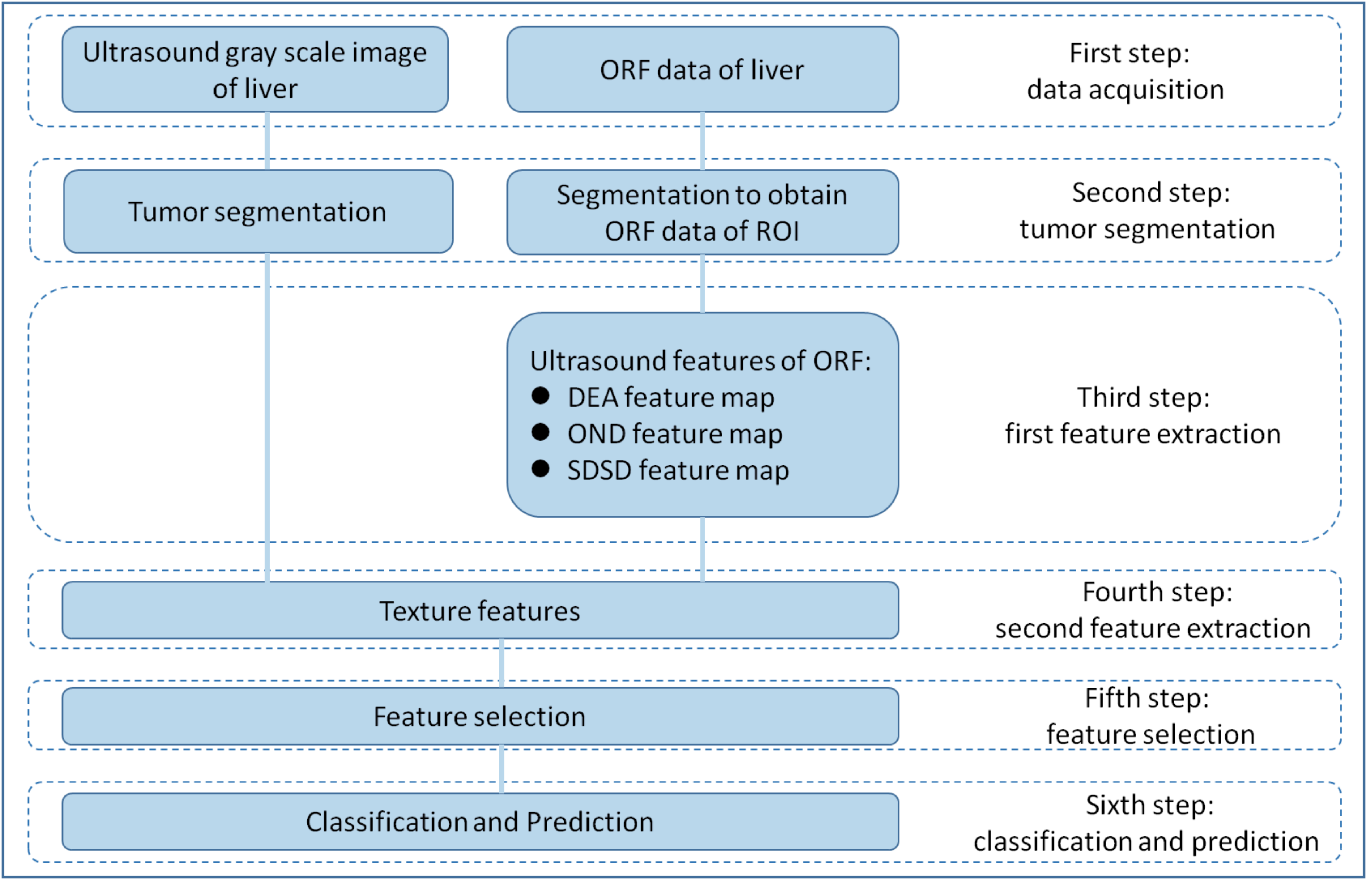
Overall design of radiomics analysis. The radiomics analysis process consisted of the following steps: (1) grayscale images and original radio frequency (ORF) data of HCC lesions obtained; (2) tumor segmentation on grayscale ultrasound images for ORF data; (3) first feature extraction to obtain three ultrasound feature maps of ORF data of region of interest (ROI); (4) second feature extraction to obtain radiomics features from three ultrasound feature maps and related grayscale ultrasound images; (5) feature selection based on sparse representation (SR) algorithm; and (6) support vector machine (SVM) classifier trained with the selected features for MVI prediction.

RA-ORF method was applied for MVI preoperative prediction. The radiomics analysis process consisted of the following steps: (1) to obtain grayscale image and ORF data of HCC lesions before operation; (2) tumor segmentation on gray scale ultrasound images of ORF data to obtain the ORF data from the region of interest (ROI) in the tumor; (3) first feature extraction to obtain three ultrasound feature maps of ORF data of ROI; (4) second feature extraction to obtain radiomics features from three ultrasound feature maps and related grayscale ultrasound images; (5) feature selection based on sparse representation (SR) algorithm (19); and (6) train support vector machine (SVM) classifier with the features sorted in step (5) to achieve further feature selection and dimension reduction, and predict MVI in patients with HCC (Figure 1).

The radiomics analysis based on ultrasound ORF signal (RA-ORF) method will be built on three ultrasound feature parameters, including direct energy attenuation (DEA), omega of Nakagami distribution (OND), and standard deviation of spectrum difference (SDSD). Leave-one-outcross-validation (LOOCV) was employed to validate the trained model.

Conventional grayscale ultrasound images will be used as the control group. The radiomics analysis for conventional ultrasound images processing included tumor segmentation, feature extraction, feature selection, and classification preoperative prediction.

All images and data were processed on MATLAB R2014b (Math Works, Inv., Natick, MA, USA).

#### Tumor Segmentation

**Figure 2:**
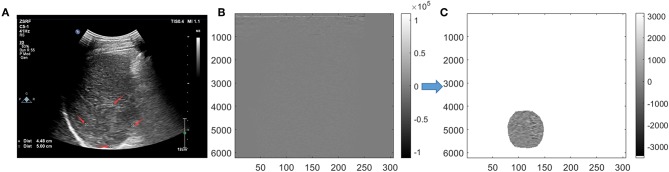
Tumor segmentation. On grayscale ultrasound image, the region of interest (ROI) was manually marked by a doctor with four white forks points **(A)**. Image were segmented in scan-line imaging way for original radio frequency (ORF) data **(B)**. After Hilbert transform and logarithmic compression of ORF signals, the grayscale ultrasound images under the scan-line images could be obtained **(C)**.

For conventional grayscale ultrasound images obtained from the first step of “data acquisition,” the ROIs were marked by an ultrasound doctor as four white forks points; then, the grayscale data of the tumor could obtained from the conventional grayscale ultrasound images by segmenting along those markers (Figure 2A).

For ORF data matrix, they were drawn directly in columns called scan-line way (Figure 2B). Data were covered with the whole picture. It is different from Figure 2A, which had values of 0 outside the sector area. Adding Hilbert transform and logarithmic compression to Figure 2B, we could get the grayscale ultrasound images in scan-line way, which clearly showed the location of the tumor. Then, segmentation was processed to obtain the location of ROI and get the ROI's ORF data. The shapes of ROI were stretched laterally at a shallow position. ROI segmented by an ultrasound doctor was used to ensure the accuracy of segmentation (Figure 2C).

#### First Feature Extraction

Feature extraction of multiparameter ultrasound features was the key step of the RA-ORF method. Three kinds of ultrasound feature parameters of ORF included time domain feature, frequency domain feature, and statistical feature and were applied.

**Figure 3:**
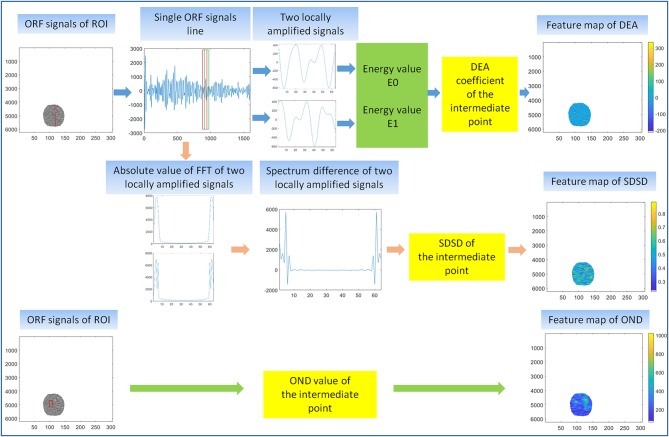
First feature extraction. The calculation principle of original radio frequency signal (ORF) signals from region of interest (ROI) in three ultrasound feature maps, including direct energy attenuation (DEA) feature map, standard deviation of spectrum difference (SDSD) feature map, and omega of Nakagami distribution (OND) feature map.

In the first feature extraction, ORF data of ROI was used to calculate three ultrasound feature parameters and further form the corresponding three ultrasound feature maps. Three ultrasound feature maps, including DEA feature map (time-domain feature), SDSD feature map (frequency-domain feature), and OND feature map (statistical feature), were established and saved in ^*^.bmp formats (Figure 3).

#### Second Feature Extraction

Second feature extraction were based on ROIs of conventional grayscale ultrasound images and the ROIs of three ultrasound feature maps obtained from ORF data. Each image can get 70 texture features: 16 features of histogram, 23 features based on gray-level co-occurrence matrix (26), 13 features based on gray-level run-length matrix (27), 13 features based on gray-level size-zone matrix (28), and five features based on neighborhood gray-tone difference matrix (29). Summary of the 70 texture features was listed in the feature extraction section of the Appendix. Then, the wavelet transformation to strip the image information layer-upon-layer by high- and low-pass filters were performed. Thereafter, four images of different frequency sub-bands and another 280 texture features could be obtained. Finally, we obtained 350 texture features from each grayscale ultrasound image and ultrasound feature maps.

### Feature Selection and Dimension Reduction

Iterative SR method were used to select key features for the classifier before classification to improve the stability of final models (30, 31). The SR coefficients of each feature were calculated by selecting part of the 42 samples in each iteration. In the SR method, the threshold Tal is set to 0.004. Then, the average SR coefficients of each feature were taken as the final SR coefficients of each feature. The importance of the features was quantified as SR coefficients. Finally, the features were sorted based on the absolute value of the final SR coefficients, and features that did not meet the threshold Tal condition were remove to achieve feature dimensionality reduction. A detailed description of SR method in feature selection is included in the feature selection section of the Supplementary Appendix.

### Classification and Prediction

SVM classifier was used in this section. Starting from number 1, the different numbers of features ranked by SR method in *Feature Selection and Dimension Reduction* were put into the SVM classifier to calculate AUC, accuracy, sensitivity, and specificity of MVI prediction in patients with HCC. We evaluated the MVI prediction models through the above parameters. The final feature dimensions of the MVI prediction models were the number of features put into the SVM classifier with the best performance in MVI prediction. This process effectively realized dimension reduction of features. Feature selection is mainly based on sparse representation, but the dimensions of features are still high after sparse representation. When implementing the classifier, the SVM uses the kernel function mapping technique to obtain the same classification result as the high-dimensional space in the low-dimensional space. In this sense, the SVM implements the further selection of features.

### Statistical Analysis

Descriptive statistics are summarized as the mean ± SD. LOOCV statistical analysis method was used to evaluate the MVI prediction models. A Tukey test, in conjunction with analysis of variance (ANOVA), was used to test the signification between any two pairs of the three ultrasound features. Receiver operating characteristic curve (ROC), precision–recall curve (PRC), and model decision curve analysis (DCA) were employed to show the overall performance of the models. Other assessment indicator included area under the ROC (AUC), accuracy, sensitivity, and specificity.

## Results

### Final Diagnosis of Patients

A total of 42 HCC patients (34 men and 8 women; age range, 23–80 years; mean, 58.5 ± 11.9 years) were finally included in our study. The surgical procedures comprised segmentectomy (*n* = 12), right anterior sectionectomy (*n* = 19), and right posterior sectionectomy (*n* = 11). The mean time between ultrasound scan and surgery was 6 days (range, 3–7 days).

Pathology data revealed the presence of MVI in 21 HCC patients as grade 1 (M1), and 21 patients were diagnosed without MVI as grade 0 (M0).

### Multiparameter Ultrasound Feature Extraction Results of ORF Signals

Multiple ultrasound parameters were extracted from ORF signals, including DEA, OND, and SDSD. They played various degrees of positive role in the MVI preoperative prediction. Compared with the M0 group, the M1 group showed larger absolute value of DEA and more serious attenuation. ANOVA analysis showed significant difference in DEA, OND, and SDSD between patient with and without MVI (*P* < 0.05).

### Second Feature Extraction and Feature Selection Results

Four pictures were included in our second feature extraction results, including grayscale ultrasound image, DEA feature map, OND feature map, and SDSD feature map. The MVI prediction model based on ultrasound grayscale image was referred to as GM. The MVI prediction model based on DEA feature map was referred to as DM. The MVI prediction model based on DEA feature map and OND feature map was referred to as DOM. The MVI prediction model based on DEA feature map, OND feature map, and SDSD feature map was referred to as DOSM.

In this texture feature extraction, we extracted 350 texture features from MVI prediction model of GM, 350 texture features from DM, 700 texture features from DOM, and 1,050 texture features from DOSM. The number of selected features of GM, DM, DOM, and DOSM MVI prediction model based SR method were 214, 253, 427, and 536, respectively.

### Diagnostic Performances of Different MVI Prediction Models

**Figure 4:**
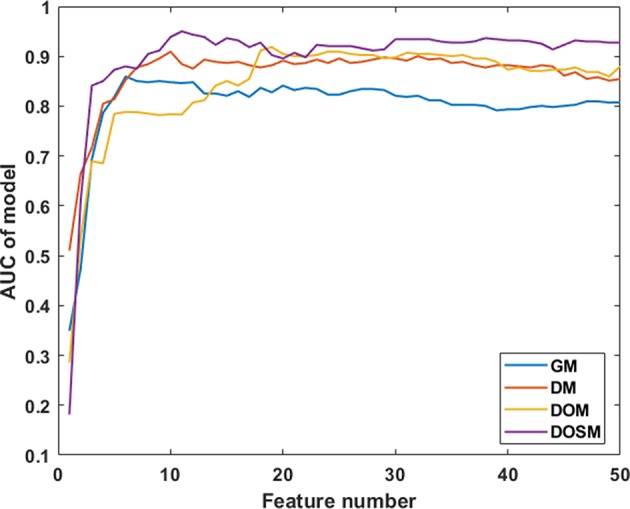
Diagnostic performances of MVI prediction models with different number of features. After feature selection, the performance of DOSM, DOM, DM, and GM models were increased gradually and maintained at a relative stable level. The changes in AUC with the increase in feature numbers were helpful to find the optimal feature dimensions of each model. The final feature dimensions of MVI prediction models of GM, DM, DOM, and DOSM were 6, 10, 19, and 11, respectively.

In SVM classifier to construct MVI prediction model, the training process of the above-mentioned model achieved further feature dimensionality reduction. Figure 4 used top 50 features after feature selection to show the performance of models utilizing different number of features. According to Figure 4, the final feature dimensions of MVI prediction models of GM, DM, DOM, and DOSM were 6, 10, 19, and 11, respectively. The maximum accuracy of the corresponding above four models by dimension reduction were 83.33, 85.71, 88.1, and 92.86%.

Table 1 shows the performance parameters of the GM model based on the conventional grayscale ultrasound images and the other three models based on the ORF signals. GM based on grayscale ultrasound image was used as a comparison test to the three MVI prediction models based on ORF signals. The AUC, accuracy, sensitivity, and specificity of GM were the lowest among the four MVI prediction models of GM, DM, DOM, and DOSM, respectively. Among the three ORF-based prediction models, the accuracy, AUC, sensitivity, and specificity of the DOSM were the highest. In the 11 selected features of DOSM, 6 features were obtained from the DEA ultrasound feature map, three features from the OND ultrasound feature map, and two features from the SDSD ultrasound feature map.

**Table 1:** Diagnostic performance of DOSM, DM, DOM, and GM for MVI classification.

**Model type**	**AUC (%, 95% CI)**	**Accuracy (%)**	**Sensitivity (%)**	**Specificity (%)**
DOSM	95.01 (0.835–0.993)	92.86	85.71	100
DOM	91.84 (0.792–0.980)	88.1	80.95	95.24
DM	90.93 (0.780–0.976)	85.71	80.95	90.48
GM	85.94 (0.717–0.947)	83.33	80.95	85.71

*AUC, area under the receiver operating characteristic curve; DOSM, MVI prediction model based on DEA feature map, OND feature map and SDSD feature map of ORF signals; DM, MVI prediction model based on DEA feature map; DOM, MVI prediction model based on DEA feature map and OND feature map; GM, MVI prediction model based on gray-scale ultrasound image*.

**Figure 5:**
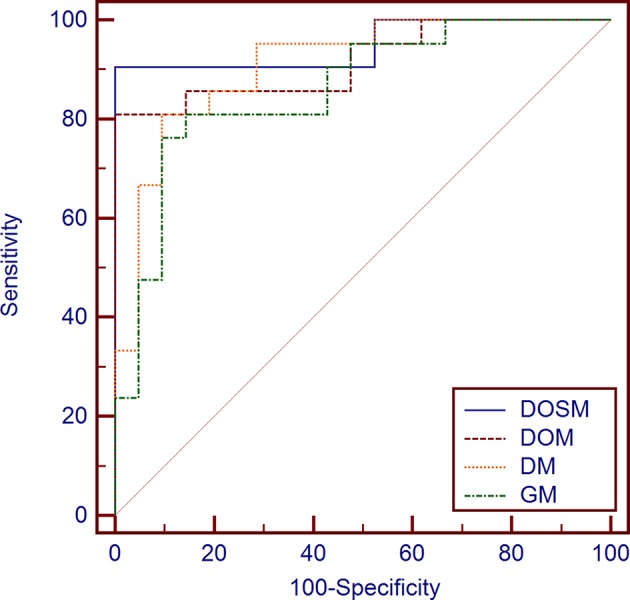
Diagnostic performances of different MVI prediction models. While comparing the AUC curves between DOSM, DOM, DM, and GM models. The AUC of DOSM (95.01%, 0.835–0.993) was the highest one among the DOSM, DOM, DM, and GM models. The AUC of GM (85.94%, 0.717–0.947) was the lowest. The AUC of DOSM is 0.95 ± 0.04, which is the highest one among the four MVI prediction models.

The AUC of DOSM (95.01%, 0.835–0.993) was the highest one among the four prediction models. The AUC of GM (85.94%, 0.717–0.947) was the lowest (Figure 5).

**Figure 6:**
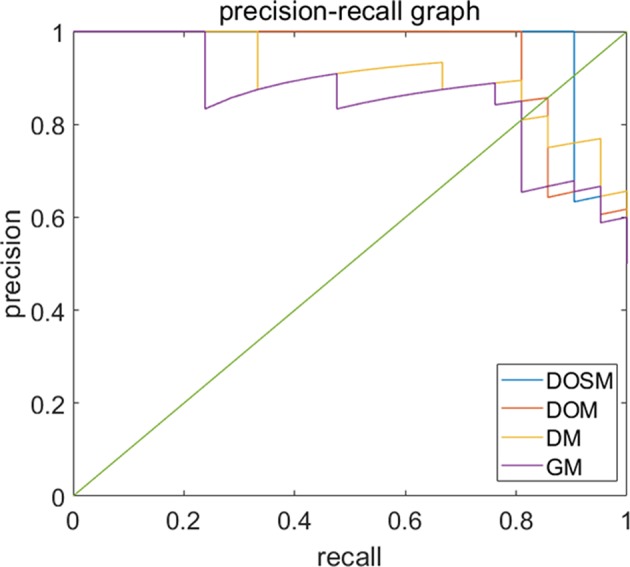
Precision-recall curves of models for prediction of MVI. Precision–recall curves (PRC) of GM, DM, DOM, and DOSM models for prediction of microvascular invasion (MVI). The DOSM based on three ultrasound feature maps selected from radio frequency signals (ORF) signals showed the best performance among GM, DM, DOM, and DOSM models in predicting the MVI classification of HCC.

Precision recall curves (PRC) of DOSM, DOM, DM, and GM are shown in Figure 6. The results showed that DOSM based on three ultrasound feature maps selected from ORF signals had more advantage compared with the other three models in predicting the MVI classification of HCC.

## Discussion

Previously, several studies proved that radiomics analysis algorithm based on ultrasound images could be helpful to extract massive features and to assist clinical decision-making. The reported ultrasound radiomics analysis algorithm based on grayscale ultrasound images, ultrasound elastography images, and contrast enhanced ultrasound images (19, 32–34). With the development of radiomics analysis, a large number of valuable features could be extracted from conventional ultrasound images, including texture features, morphological features, and some other specific features (35, 36). However, conventional ultrasound images might be affected by post-processing procedure; as a result, they will lose a lot of useful information compared with ORF signals (21–24). The radiomics analysis technology based on ORF data was applied in our present study. We extracted three ultrasound feature maps of ORF signal of HCC lesions, combining with the iterative SR method and SVM classifiers to reduce the feature dimensions and build MVI prediction model. In our results, 11 highly correlated radiomics features were finally obtained to establish an effective MVI prediction model of DOSM. DOSM prediction model based on RA-ORF showed superior performance for MVI prediction, which make full use of the advantages of signal processing technology. It could extract more useful radiomics features and improve the accuracy of MVI classification.

Previously, several studies tried to classify diseases by ORF signal combined with radiomics analysis (34, 37) to prove that time-domain features (38), statistical distribution features, and frequency-domain features (39) of ultrasound ORF signals be helpful in disease recognition (40). In signal processing, the ultrasound feature parameters of DEA, SDSD, and OND, which were obtained from ORF signals in time, frequency, and statistics domains, always have clear and valuable physical significance. DEA of time-domain characteristics of ORF signals represents the direct energy attenuation in ROI. When the normal tissue changes, its microstructure will change accordingly, which leads to the change in attenuation. SDSD of frequency-domain characteristics of ORF signals represents standard deviation of spectrum difference, which is a common parameter to reflect spectrum differences between tissues in spectrum analysis. OND of statistical characteristics of ORF signals represents omega of Nakagami distribution of ROI. The parameter values of Nakagami distribution for the second harmonic envelope signals from different degrees of non-linearity in tissue are significantly different. According to this, we can quantitatively analyze the difference in non-linear characteristics between normal and diseased biological tissue (41). At present, advanced radiomics method makes it possible to extract huge amounts of features and to select valuable features from multiclass ultrasound feature maps consisting of DEA, SDSD, and OND. In our results, ROC and PRC curves both validated the reliability of DOSM model in MVI prediction of HCC lesions. Our RA-ORF method combined ORF-based signal processing technology with radiomics analysis, which showed a good classification performance on MVI prediction. Among the three ORF-based prediction models, the accuracy, AUC, sensitivity, and specificity were gradually improved. Some valuable radiomics features were further extracted for MVI prediction. Meanwhile, the performance of MVI prediction models in HCC lesions was improved accordingly. The radiomics algorithm based on ORF signal was superior to that based on conventional grayscale ultrasound images.

Pathologically, MVI is defined as the presence of micrometastatic HCC emboli within the vessels of the liver (9). Relevant studies have shown that there is a correlation between tissue microstructures and spectrum feature (42). Spectrum analysis based on ORF signals can obtain abundant microstructural information, which might be completely lost in conventional grayscale ultrasound images (21–24, 42). Therefore, by extracting frequency-domain features and combining radiomics analysis, different pathological tissues could be analyzed. The presence of MVI in HCC lesions may cause changes in tissue attenuation coefficient accordingly. It is possible for us to use the time-domain features of DEA calculated from radiomics analysis of ORF signals to predict MVI in HCC lesions. The DOSM prediction model based on RA-ORF in our study reached sensitivity of 85.71%, specificity of 100%, and AUC of 95.01%. It was proved to be superior to DOM, DM, and GM models. Our initial results showed that the AUC of the DM model based on RA-ORF, which uses time-domain features of DEA, was better than the GM model based on RA-USI with conventional grayscale ultrasound images.

Our study has several limitations: the patient number is relatively limited; only three ultrasound parameters of DEA, OND, and SDSD based on ORF signals were included. The stability evaluation of RA-ORF based radiomic analysis would be further improved by multicenter studies in the future.

## Conclusion

In conclusion, radiomics algorithm based on RA-ORF and SAP technology might provide useful information for preoperative MVI prediction in HCC lesions. Depending on the unique advantages of ultrasound imaging such as real-time imaging, low cost, and no radiation exposure risk, it might be a promising method in future clinical application.

## Data Availability Statement

All datasets generated for this study are included in the article/Supplementary Material.

## Ethics Statement

The studies involving human participants were reviewed and approved by Zhongshan Hospital, Fudan University. The patients/participants provided their written informed consent to participate in this study.

## Author Contributions

Each author had participated sufficiently in the paper and approved the manuscript for submission. W-PW and JY contributed to this paper with conception and design of the study. YD and QL performed literature review and analysis. YD and QZ performed the clinical ultrasound scan. LY-L, ZY, and MD conducted the image analysis. YD and Q-MW contributed to drafting and critical revision and editing, and all authors gave final approval of the final version.

### Conflict of Interest

The authors declare that the research was conducted in the absence of any commercial or financial relationships that could be construed as a potential conflict of interest.

## Abbreviations

ORF: Original radio frequency signals
MVI: Microvascular invasion
HCC: Hepatocellular carcinoma
RA-ORF: Radiomics analysis method based on ultrasound original radio frequency signal
ROI: Region of interest
SR: Sparse representation
SVM: Support vector machine
LOOCV: Leave-one-out cross-validation
DEA: Direct energy attenuation
OND: Omega of Nakagami distribution
SDSD: Standard deviation of spectrum difference
SAP: Signal analysis and processing
DM: Microvascular invasion prediction model based on direct energy attenuation
DOM: Microvascular invasion prediction model based on direct energy attenuation and omega of Nakagami distribution
DOSM: Microvascular invasion prediction model based on direct energy attenuation, omega of Nakagami distribution and standard deviation of spectrum difference
AUC: Area under curve
MRI: Magnetic resonance imaging
CT: Computed tomography
RA-USI: Radiomics analysis based on conventional ultrasound image
ANOVA: Analysis of variance
ROC: Receiver operating characteristic curve
PRC: Precision recall curve
DCA: Decision curve analysis.
