# Supplementary material for: Preoperative Prediction of Microvascular Invasion of Hepatocellular Carcinoma: Radiomics Algorithm Based on Ultrasound Original Radio Frequency Signals
Source: Front Oncol. 2019 Nov 14;9:1203. doi: 10.3389/fonc.2019.01203 (PMC6868049; doi:10.3389/fonc.2019.01203)
Supplement: Supplementary file 1 [file Data_Sheet_1.docx]

# Appendix

## Feature extraction

| Summary of the texture features | | | |
| --- | --- | --- | --- |
| Feature type | Feature name |  | Feature number |
| Histogram | 1) Energy 2) entropy 3) Tkurtosis 4) mean 5) MAD 6) media 7) Range 8) uniformity 9) variance 10) RMS 11) skewness 12) deviation 13) kurtosis 14) mean 15) variance 16) skewness |  | 16 |
| GLCM | 17) Energy 18) entropy 19) dissimilarity 20) contrast 21) inversed difference 22) correlation 23) homogeneity 24) autocorrelation 25) cluster shade 26) cluster prominence 27) maximum probability 28) sum of squares 29) sum average 30) sum variance 31) sum entropy 32) difference variance 33) difference entropy 34) information measures of correlation1 35) information measures of correlation2 36) maximal correlation coefficient 37)maximal correlation coefficient 38) inverse difference normalized 39) inverse difference moment normalized |  | 23 |
| GLRLM | 40) Short-run emphasis 41) long-run emphasis 42) gray-level nonuniformity 43) run-length nonuniformity 44) run percentage 45) low gray-level run emphasis 46) high gray-level run emphasis 47) short-run low gray-level emphasis 48) short-run high gray-level emphasis 49) long-run low gray-level emphasis 50) long-run high gray-level emphasis 51) gray-level variance 52) run-length variance |  | 13 |
| GLSZM | 53) Small zone emphasis 54) large zone emphasis 55) gray-level nonuniformity 56) zone-size nonuniformity 57) zone percentage 58) low gray-level zone emphasis 59) high gray-level zone emphasis 60) small zone low gray-level emphasis 61) small zone high gray-level emphasis 62) large zone low gray-level emphasis 63) large zone high gray-level emphasis 64) gray-level variance 65) zone-size variance |  | 13 |
| NGTDM | 66) Coarseness 67) contrast 68) busyness 69) complexity 70) strength |  | 5 |

## Feature selection

SR-based feature selection identifies the most relevant features according to the importance index generated by each iteration. An iterative process can be expressed as:

$$\hat{d}^{(k)}={argmin}_{d}\left\| s^{(k)}-F^{(k)}d \right\|_{2}^{2}+\varepsilon\left\| d \right\|_{0}$$

Where $S^{(K)}$ is the label used for the k-th iteration; $F^{(k)}$ is the feature selected for the k-th iteration; $\varepsilon$ is a small constant; $\hat{d}^{(k)}$ is the coefficient calculated by the k-th iteration. The average of $\hat{d}^{(k)}$ for the k-th iteration can be calculated as:

$$d^{(k)}=\frac{1}{k}\sum_{i=1}^{k} \hat{d}^{(k)}$$

The $d^{\left( k \right)}$used for feature selection was the features’ scores through the averaging operation of k itarations.

The higher the score, the more importance of the feature. In this way, In this way, we can sort the importance of features and obtain the feature selection results.
